# Comparative Evaluation of the Effect of Photobiomodulation on Pain Reduction in Individuals Undergoing Segmental Retraction Using a Closing Loop: A Randomized Controlled Clinical Trial

**DOI:** 10.7759/cureus.58001

**Published:** 2024-04-10

**Authors:** Tanvi A Khot, Priya Lele, Vidya Dodwad, Amol Patil, Nishita Bhosale, Manasi Yewale

**Affiliations:** 1 Periodontology, Bharati Vidyapeeth Dental College and Hospital, Pune, IND; 2 Orthodontics and Dentofacial Orthopaedics, Bharati Vidyapeeth Dental College and Hospital, Pune, IND

**Keywords:** split-mouth, pbmt, pain control, laser, photobiomodulation

## Abstract

Background: Pain following orthodontic treatment is a common reason for apprehension and treatment discontinuation. Research on modalities to control pain in orthodontic patients has gained special attention. Low-level laser therapy (LLLT) is studied as an alternative pain management modality free of the adverse effects of analgesic medications.

Objectives: This study evaluated the effectiveness of photobiomodulation therapy (PBMT) for pain control following the activation of a closing loop for canine retraction.

Method: This is a split-mouth, placebo-controlled, single-blinded randomized clinical trial that evaluated 16 patients who need canine retraction using closing loops. Two maxillary quadrants were allotted into test and control groups using the coin toss method. In the test group, a low-intensity laser with 810 nm wavelength for 60 seconds in pulsated non-contact mode was used in the buccal, palatal, mesial, and distal regions of the canine immediately after activating the loop. The control site received placebo radiation. The pain level was recorded 2, 24, 48, and 72 hours after intervention in the control and test groups using the Visual Analogue Scale (VAS). The test and control groups were compared using Student's t-test. A p-value ≤0.05 was considered statistically significant. Analyses were conducted using IBM SPSS Statistics for Windows, Version 25.0 (Released 2017; IBM Corp., Armonk, New York, United States).

Result: Both groups had a significant statistical difference in the pain score. The laser group showed a statistically significant lower pain score compared to the control group at all time points.

Conclusion: Photobiomodulation by 810 nm 300 mW diode laser can effectively reduce pain following the retraction of maxillary canines.

## Introduction

Orthodontic treatment is required in some patients to enhance their masticatory function and smile aesthetics. Orthodontic tooth movement results from bone and periodontal tissue remodeling in response to mechanical forces. Through bone resorption and apposition, orthodontic force encourages tooth displacement in the periodontal ligament space, which results in bone remodeling in the alveolus. This process often causes pain, induced by factors like alterations in the blood flow, the release of inflammatory cytokines, stimulation of afferent A-delta and C nerve fibers, hyperalgesia, and neuropeptides.

Pain is an undesirable drawback of tooth movement that causes concern and discomfort for the patients. Approximately 90% of patients feel pain during the first phases of orthodontic treatment [1], which reduces patient collaboration and affects speech, mastication, treatment course, and patient cooperation. Also, orthodontic pain usually starts two hours after force application, reaches a peak level at 24 hours, and stays for approximately five days. The patients characterize this pain as discomfort, dull pain, or hypersensitivity in the affected teeth. Despite having a significant clinical benefit, orthodontic discomfort is frequently disregarded and undervalued [2]. The major mediators responsible for pain are prostaglandin, substance P, bradykinins, encephalin, leukotrienes, and histamines due to their role in sensitizing nerve endings and aggravating inflammation. The primary inflammatory mediators are the cytokines such as the chemokine IL-8, which brings about the recruitment and chemotaxis of neutrophils, which explains its high concentration at tissue injury sites.

Canine retraction is an essential procedure in patients requiring space closure. However, the load system, including the moment-to-force ratio, can be affected by the changes in canine angulation and interbracket distance. This heavy load system, including the moment-to-force ratio, is known to cause intense pain and discomfort to the patient after every activation. This resultant pain is managed conventionally with nonsteroidal anti-inflammatory drugs (NSAIDs) which decrease the prostaglandin synthesis. However, NSAIDs decelerate orthodontic tooth movement and are associated with side effects like gastrointestinal problems, allergic reactions, headache, nephrotoxicity, thrombocytopenia, hepatic toxicity, etc. Other pain reduction methods include vibratory stimulation, transcutaneous electrical nerve stimulation, and chewing gum. However, there is limited literature due to scant evidence and unclear influence [3]. Moreover, masticating hard objects might aggravate pain and cause discomfort [4].

Recently, photobiomodulation therapy (PBMT), also known as low-level laser therapy (LLLT), is gaining popularity as a pain modulation therapy during orthodontic tooth movement. The PBMT pain attenuation is attributed to its inhibitory effect on nerve depolarisation (C fibers), ATP synthesis, and reduction in prostaglandin levels [3]. Photobiomodulation is the direct application of laser light to stimulate cell response (biomodulation) which contributes to its biostimulatory effect on wound healing, muscle relaxation, nerve regeneration, collagen synthesis, fibroblast proliferation, acceleration in bone regeneration, reduction in inflammation, improvement of blood circulation, and increase in cell activity. LLLT has a low-energy output and hence does not cause temperature elevation of the local tissue above normal temperature [4].

This study hypothesizes that a significant influence on the load system delivered to the tooth causing pain in one specific area will be caused by clinical changes in canine positions and angulation when retracting.

Since there are few studies to investigate a single dose of LLLT on segmental canine retraction during the activation of the closing loop, the present study aimed to evaluate a single dose of LLLT on orthodontic pain management during canine retraction.

## Materials and methods

This is a split-mouth, randomized clinical trial that consisted of 16 patients who were referred to the Orthodontics Department of Bharati Vidyapeeth Dental College and Hospital in Pune, India. It was a single-blinded study: patients and orthodontists were blinded to the allocations, but the laser operator (periodontist) was not blinded.

Before starting the study in August 2022, approval was obtained from the Institutional Ethics Committee of Bharati Vidyapeeth Dental College and Hospital (approval number: EC/NEW/INST/2021/MH/0029). The Clinical Trials Registry-India (CTRI) trial reference number is REF/2024/03/080888. 

Written informed consent was procured from all patients (or parents/legal wardens for participants less than 18 years of age).

In this present study, the canine retraction was the first procedure after the extraction of the first premolar. The patients did not undergo or have any experience of any orthodontic treatment before the canine retraction.

Selection criteria

Inclusion criteria included the absence of any systemic disease, periodontal/periapical disease, no history of previous orthodontic treatment, good oral hygiene, and the willingness to participate in the study. Exclusion criteria included intake of analgesics and anti-inflammatory medications during the study, presence of chronic pain, facial neuralgia, gingival inflammation, gingival pigmentation, pregnancy or nursing, dental or gingival pain before the treatment onset, and ankylosis.

All the patients were prospectively recruited from the Orthodontics Department of Bharati Vidyapeeth Dental College and Hospital, Pune. Participants were recruited from 01/10/2022 to 15/04/2023.

Experimental design

**Figure 1:**
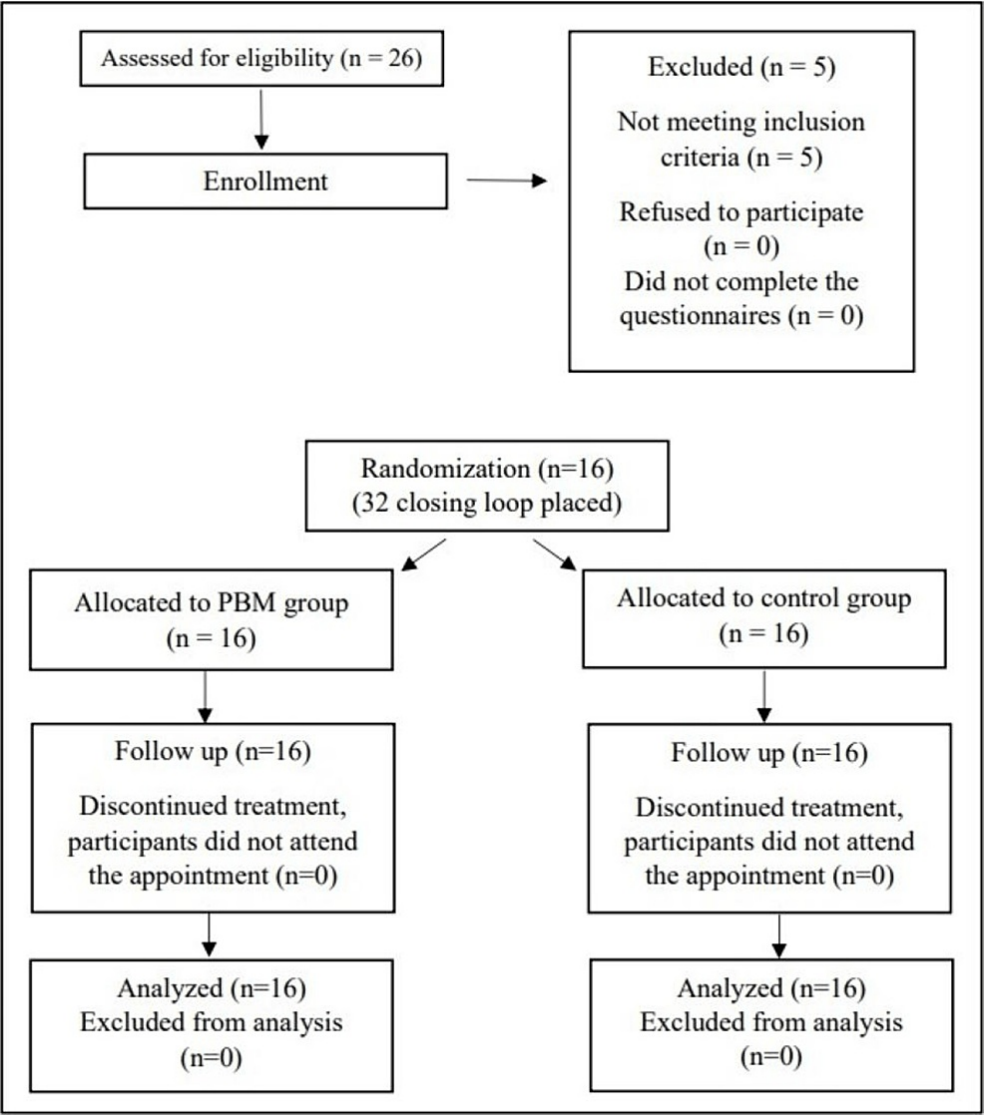
CONSORT flow diagram representing the study design of the trial CONSORT: Consolidated Standards of Reporting Trials

The present study is a split-mouth, single-blinded, placebo-controlled, randomized clinical trial. The study design has received an institutional ethics committee's approval. No changes occurred in the methodology after the commencement of the trial. A single examiner selected patients based on a questionnaire and clinical evaluation to ensure strict adherence to the inclusion and exclusion criteria. Figure 1 shows the patients' flow through the trial, where n is the number of participants. There were neither dropouts nor exclusions once the trial commenced.

Uniform treatment protocols

The orthodontic treatment plan consists of the extraction of maxillary premolars for space making or for the treatment of maxillary protrusion. Metal pre-adjusted brackets of 0.018 slots were used. After banding and bracket bonding, the stages for aligning and leveling were carried out by beta-titanium archwires of diameters 0.016x0.022 in, and canine retraction began using 0.018-in-stainless steel wires. The second molars were banded and inserted into wires on both sides, to maintain the anchorage.

The coin toss method divided the quadrants into control and test groups. 

Loop placement

A clinician from the Orthodontics Department placed the closing loop using the stainless steel wire for the retraction of canines in patients with highly placed canines.

Laser application

After activating the closing loop, participants received PBMT in one quadrant for pain control and placebo treatment in the other quadrant. 

**Figure 2:**
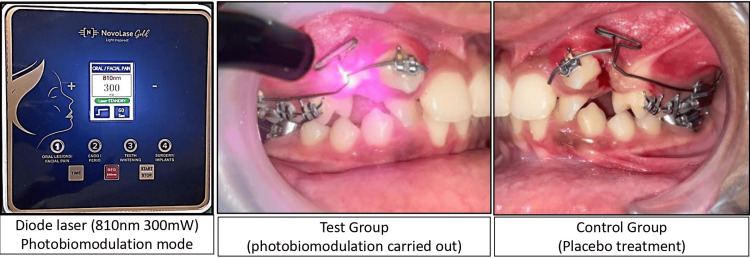
Diode laser with 810 nm wavelength and 300 mW power carried out in the test group and placebo treatment given in the control group

The laser in this study is NovoLase Gold (diode laser) (wavelength of 810 nm with 300 mW power) (as per the manufacturer's guidelines) carried out in non-contact mode, 1 cm away from the concerned site, at the canine, second premolar, and first molar region around the tooth surface for 30 seconds per tooth each on the buccal and lingual aspects (a total of 180 seconds). Laser-related procedures were carried out by the same operator (Figure 2).

Participants were instructed to avoid taking any medications during the experimental period; otherwise, they would be excluded from the study.

Abiding by the biosafety rules, the patients and the doctor were given safety glasses during laser irradiation. For the control group to ensure patient blinding, the same acoustic sounds were recreated by the laser.

The main outcome of the present study was to evaluate the reduction of pain intensity induced by PBMT compared to placebo treatment.

All individuals were asked to rate their pain intensity on a 10 cm Visual Analogue Scale (VAS) at the test and control sites at 2, 24, 48, and 72 hours after the activation of the closing loop. A score of 0 indicates no pain, whereas a score of 10 is considered as utmost pain possible. 

Statistical analysis

IBM SPSS Statistics for Windows, Version 20.0 (Released 2011; IBM Corp., Armonk, New York, United States) is used to analyze the data. All the quantitative data will be tabulated using means and standard deviations. Data will be tabulated using numbers and percentages. Comparison among the two groups will be done using Student's t-test (if the data is parametric) and Wilcoxon signed-rank test (if the data is non-parametric).

Descriptive statistics have been used for the expression of means and standard deviations. The VAS scores in the test and control groups at different time intervals were compared using the repeated measures ANOVA test for pairwise comparisons. At each period, the comparison between the test and control groups was done using Student's t-test. In the above tests, a p-value less than or equal to 0.05 was considered statistically significant. All the analyses were conducted using IBM SPSS Statistics for Windows, Version 25.0 (Released 2017; IBM Corp., Armonk, New York, United States).

The significance for all statistical tests was predetermined at p<0.05.

## Results

Patients were evaluated to assess their pain perception after the application of LLLT. Table 1 shows the details of the patients who participated in the study.

**Table 1:** Details of patients participating in the study A total of 16 participants were included in the study with an age range from 15.5 to 23.5 years

Total participants	16
Males	10
Females	6
Age (years)	15.5-23.5

**Figure 3:**
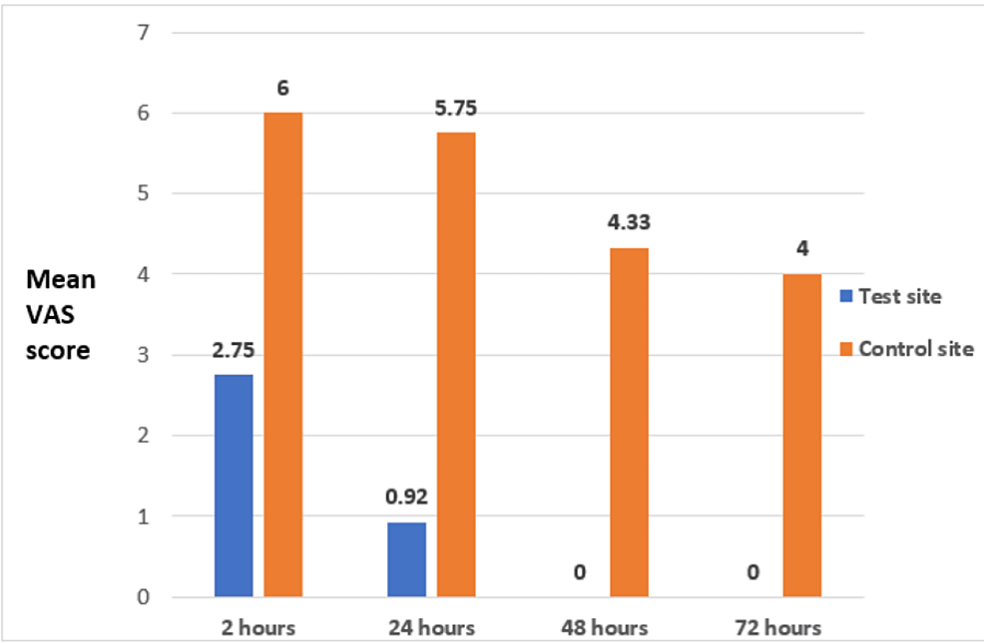
Comparison of the VAS scores for the pain intensity at the control and test sites for individuals undergoing segmental retraction at various time intervals VAS: Visual Analogue Scale

For evaluating pain perception, the VAS score data was combined into irradiated and non-irradiated groups, in the different periods of analysis (2, 24, 48, and 72 hours). Figure 3 shows the observation that the level of pain perception was significantly lower on the irradiated side, in each experimental time of 2, 24, 48, and 72 hours.

## Discussion

A major drawback of orthodontic treatment is pain and discomfort following force application. Laser therapy, for example, has been used to reduce pain and the duration of treatment. Photobiomodulation referred to as LLLT is currently demanded as a pain reduction therapy. LLLT stands for low-power laser, biostimulation, therapeutic laser, soft tissue laser, and cold laser [5]. Laser biomodulation is based on the Arndt-Schulz law. According to this law, a small dose of any substance has a stimulating effect, whereas a higher dose has an inhibitory effect. LLLT is a non-invasive and easy-to-apply technique that encounters only a limited side effect. There have been many studies on LLLT, and most state that it is effective in controlling pain; however, a few studies found results to the contrary. The wide variation in the experimental designs and aspects like wavelength, power output, energy density, mode, duration, and frequency of laser could be the reason for the non-congruous conclusions and present a challenge that needs to be negotiated. PBMT has recently been suggested as an effective orthodontic analgesia treatment due to its benefits in analgesia and biostimulation with limited side effects. The efficacy of PBMT can be influenced by factors including light source, frequency, power output, wavelength, spot size, energy density, mode of operation (continuous or pulsed wave), time of exposure, and application interval. PBMT has been demonstrated to be effective in pain management in several orthodontic procedures, like separator placement, canine retraction, and both the initial and final stages of archwire placement [6].

In the present study, pain intensity caused by canine retraction was measured using the VAS at 2, 24, 48, and 72 hours to test the effect of photobiomodulation on the test site as our primary aim. The null hypothesis was partially rejected. Results showed a significant decrease in pain in the test site compared to the non-laser application control site.

Valuating subjective phenomena like pain is a challenge. Hence, a reliable way to assess localized pain in a split-mouth format is to evaluate the pain caused by canine retraction. However, due to the study design difficulties, only three studies have evaluated the pain of canine retraction, on 12, 20, and 30 patients [6-8]. The study hypothesizes that clinical alterations in canine positioning and angulation due to the retraction of the canine will have a significant impact on the load system delivering pain to the tooth.

NovoLase Gold (diode laser) is used in the present study with a wavelength of 810 nm with 300 mW power. Different laser wavelengths have been used in previous studies ranging from 600 nm to 1000 nm with an energy density of 0.04-60 J/cm^2^ with diode laser, Ga-Al-As diode laser, or He-Ne laser. Different authors used different laser wavelength radiation and obtained acceptable results for pain reduction. Yoshida et al. used 880 nm wavelength, Sobouti et al. used 790 nm wavelength, and Tortamano et al. used 810 nm wavelength [9-11]. Most studies evaluated pain invoked by local separator placement, which cannot simulate common orthodontic pain caused by real tooth movements. A few studies have used activated archwires to cause widespread orthodontic pain; however, this approach prevents the use of split-mouth designs that are functional and have appropriate contrasts between both sides of the mouth [12,13].

Researchers observed that the 810 nm laser was found to be the most effective [14]. Similar to the present study, Farias et al., Eslamian et al., and Youssef et al. found a reduction in orthodontic pain using an 810-wavelength laser for 15 seconds per point [15-17]. Bayani et al. from a randomized controlled trial study concluded that single irradiation from LLLT is the best strategy for orthodontic pain control [14]. Verschueren et al. determined that light-induced light therapy (LLLT) produces a photobioactive reaction to promote cellular proliferation and differentiation [18]. This, in turn, increases local blood circulation, eliminates inflammatory mediators that cause pain, and improves cellular activities [12]. Brito et al. in 2022 carried out a single-dose irradiation therapy using an infrared laser of 808 nm wavelength on all teeth for patients undergoing non-extraction orthodontic therapy, compared pain perception with the control site, and reported a significantly shorter duration of pain and lower pain score in the laser group compared to the control group [19]. In a study by El-Bialy et al., on the application of an 810-nm-wavelength infrared laser once a week on maxillary molars during distalization, patients reported significantly lesser pain in the LLLT group for the first three days [20]. Another study showed a statistically significant difference between the control (placebo) group and the irradiated group [21]. However, contradicting results were seen in three studies which do not show any significant difference in pain relief in orthodontic patients following the use of laser therapy [22-24]. In the study conducted by AlSayed Hasan et al. [23], the mean pain scores in the LLLT group were less than the scores of the placebo group. This suggests some clinical efficiency of PBMT even in the absence of statistical significance.

Limitations

Laser can be irradiated at multiple intervals, and more studies need to be carried out using a larger sample size to determine the pain control parameters accurately. 

## Conclusions

The present study suggests pain reduction with reduced consumption of analgesics with the use of LLLT as an aid to reduce pain. LLLT has a brief history and strong evidence, which supports its use in pain management. Successful outcomes depend on good clinical skills with an understanding of the nature of pain and the mechanism of the laser. Our results promote the use of LLLT to reduce pain with minimal side effects. 
